# Development and Validation of QuEChERS Followed by UHPLC-ToF-MS Method for Determination of Multi-Mycotoxins in Pistachio Nuts

**DOI:** 10.3390/molecules26195754

**Published:** 2021-09-23

**Authors:** Ana Rita Soares Mateus, Sílvia Barros, Angelina Pena, Ana Sanches Silva

**Affiliations:** 1 Faculty of Pharmacy, University of Coimbra, Polo III, Azinhaga de Stª Comba, 3000-548 Coimbra, Portugal; anarsmateus@hotmail.com (A.R.S.M.); ana.silva@iniav.pt (A.S.S.); 2 National Institute for Agricultural and Veterinary Research (INIAV), I.P., Rua dos Lagidos, Lugar da Madalena, 4485-655 Vairão, Vila do Conde, Portugal; silvia.barros@iniav.pt; 3 LAQV, REQUIMTE, Laboratory of Bromatology and Pharmacognosy, Faculty of Pharmacy, University of Coimbra, Polo III, Azinhaga de Stª Comba, 3000-548 Coimbra, Portugal; 4 Center for Study in Animal Science (CECA), ICETA, University of Oporto, Apartado, 55142 Oporto, Portugal

**Keywords:** mycotoxins, pistachios, *Pistacia vera* L., validation, ultra-high performance liquid chromatography, time of flight mass spectrometry, EMR-lipid, z-Sep

## Abstract

Pistachios are one of the types of tree nut fruits with the highest mycotoxin contamination, especially of aflatoxins, worldwide. This study developed a Quick, Easy, Cheap, Effective, Rugged, and Safe (QuEChERS) method that was followed by Ultra-High Performance Liquid Chromatography combined with Time-of-Flight Mass Spectrometry (UHPLC–ToF-MS) for the determination of mycotoxins in pistachios. Different approaches to dispersive solid phase extraction as a clean-up method for high lipid matrices were evaluated. For this, classic sorbents such as C18 (octadecyl-modified silica) and PSA (primary secondary amine), and new classes of sorbents, namely EMR-Lipid (enhanced matrix removal-lipid) and Z-Sep (modified silica gel with zirconium oxide), were used. The QuEChERS method, followed by Z-Sep d-SPE clean-up, provided the best analytical performance for aflatoxins (AFB1, AFB2, AFG1 and AFG2), ochratoxin A (OTA), zearalenone (ZEA), toxin T2 (T2) and toxin HT-2 (HT2) in pistachios. The method was validated in terms of linearity, sensitivity, repeatability, interday precision and recovery; it achieved good results according to criteria imposed by Commission Regulation (EC) no. 401/2006. The method was applied to real samples and the results show that pistachios that are available in Portuguese markets are safe from mycotoxins that are of concern to human health.

## 1. Introduction

Pistachio (*Pistacia vera* L.)*,* as other tree nut fruits like hazelnut, almonds, walnuts and cashew nuts, are characteristic of Mediterranean diet [1], consumed as a snack (raw, roasted or salted) but is also used in ice cream and bakery. Consumption of pistachio has been increasing due to recognized nutritional quality they are low in calories, high in monounsaturated fatty acids and low in saturated fatty acids. In addition, they are a good source of proteins, carbohydrate and dietary fibres, vitamins (A, E, K, B1 and B6) and minerals (potassium, phosphorus, magnesium, and iron). Also, several health benefits are associated with pistachio consumption, studies show reduction of the risk of coronary heart disease [2], reduction of oxidative stress and inflammation, blood glucose control, improved appetite management and consequent better weight control [3]. Besides pleasant flavour, these health benefits turn pistachio in one of the most popular nuts in the world. In 2019, the global market of pistachio was dominated by Iran and United States of America, which produced 337,000 tons and 335,000 tons, respectively, followed by China and Turkey [4,5].

Mycotoxins are secondary metabolites of filamentous fungi, constituting a heterogenous group of molecules with low molecular mass [6] that can contaminate pistachio nuts. Fungal contamination occurs along food chain, in pre- and post-harvest (drying, transport, storage, and processing) when environmental conditions, such as temperature and relative humidity, are favorable to its growth. Also, crop damage due to insect infestation or mechanical damage are factors influencing fungal contamination. Food may be contaminated with a number of different mycotoxins, when conditions are favorable for fungal contamination, more than one fungal species can contaminate food, and a single species of fungi can produce several toxic metabolites [7,8]. However, the presence of fungi may not be related to the presence of mycotoxins because, on one hand, not all fungi are mycotoxin producers (i.e., toxigenic), and, on other hand, mycotoxins are only produced under certain conditions. One of the conditions that contribute for the production of mycotoxins by fungi is the competition among different fungi species [9].

Mycotoxins are a concern for food safety, FAO estimates that 25% of foods are contaminated by mycotoxins, with consequences on health and economy [7,10]. Major mycotoxins like aflatoxins (AFB1, AFB2, AFG1 and AFG2), ochratoxin A (OTA), fumonisins (FB1 and FB2), zearalenone (ZEA), toxin T2 (T2), toxin HT-2 (HT2) and desoxynivalenol (DON) may cause adverse health effects, mostly due to chronic exposure, like carcinogenicity, mutagenicity, teratogenicity, cytotoxicity, neurotoxicity, nephrotoxicity, immunosuppression and estrogenic effects [11]. According to International Agency for Research on Cancer (IARC), aflatoxins are included in group 1 (carcinogenic to humans), FB1, FB2 and OTA are included in the group 2B (possibly carcinogenic to humans), and ZEA and T2 are included in group 3 (not classifiable as to its carcinogenicity to humans) [12,13] Although those of group 3 are considered not carcinogenic, they can cause other adverse effects. In addition, synergies can also occur among toxic effects of mycotoxins [7].

Despite pistachios have been associated to health benefits, they can be seen as an important source of exposure to mycotoxins, especially aflatoxins, are due to division of bark at end of maturation [14,15], constituting a current public health problem. To ensure consumer’s health, occurrence of mycotoxins is monitored, and maximum levels are regulated worldwide. In the Europe Union (EU), Regulation no. 1881/2006 and its amendments establishes the maximum levels of mycotoxins in nuts. Based in their toxicity and occurrence, a limit of 4 μg/kg is provided for total aflatoxins (sum of AFB1, AFB2, AFG1 and AFG2) and a limit of 2 μg/kg for AFB1, which is the mycotoxin representing the greatest concern due to its carcinogenicity [16]. In Codex Alimentarius, the maximum levels for total aflatoxins in pistachios for human direct consumption are 10 μg/kg. Though, maximum levels of DON, FB1, FB2 and OTA in treenuts are not established [17]. European legislation also covers other mycotoxins, for OTA there is a suggestion to fix a maximum level in dried fruit other than raisins. Concerning to DON, ZEA, FB1, FB2, T2 and HT2 no references of the maximum levels in tree nut fruits are present, because, so far, there is no significant reported occurrence.

The determination of mycotoxins in pistachio is challenging because, on one hand, mycotoxins are present in low concentrations and are distributed heterogeneously, and on the other hand, pistachio are complex food matrix due to their multiple components and high lipid content (53%) [18,19]. Analysis of mycotoxins follows a common protocol: sampling, sample preparation, extraction, with or without purification step and detection/quantification [20]. Firstly, extraction of mycotoxins from the solid matrix to a liquid phase is generally performed with QuEChERS (Quick, Easy, Cheap, Effective, Rugged and Safe) method, allowing separation of a wide range of analytes and analysis of several samples in a short time (high throughput) and uses a small amount of sample and low volume of solvents. QuEChERS extraction is divided in two stages: (1) extraction step based on salting-out effect, using acetonitrile (ACN) and combination of magnesium sulfate (MgSO_4_) with sodium chloride (NaCl) in 4:1 ratio as extraction salts and (2) clean-up with adsorbents to remove interferers, usually by dispersive Solid Phase Extraction (d-SPE). Sorbents like octadecyl modified silica (C18) or primary secondary amine (PSA) are known as the classical sorbents for clean-up step in high lipid matrix.

However, new classes of sorbents have recently appeared on the market as Enhanced Matrix Removal-Lipid (EMR-Lipid) and Z-Sep or Z-Sep^+^. EMR-Lipid, which details of the structure are unknow, provides good reduction of co-extracted matrix compounds, with good recoveries in different samples [21,22,23,24,25]. Z-Sep is based on modified silica gel with zirconium oxide and Z-Sep^+^ consists of both zirconia and C18 dual bonded on the same silica particles. These sorbents are more selective to remove fat and pigments from sample extracts, with greater analyte recovery [19,21]. So, these new two sorbents show potential to multi-mycotoxin clean-up in pistachio samples.

Nowadays, for detection/quantification, liquid chromatography coupled with mass spectrometry (LC-MS) is the gold standard for multi-class mycotoxins, with high sensitivity and specificity, low limits of detection and quantification, and good accuracy [26]. Multi-mycotoxin methods can determine a greater number of mycotoxins in a single chromatographic run, very important due to mycotoxins’ co-occurrence.

The Rapid Alert System for Food and Feed (RASFF) reported, in 2019, 588 notifications for mycotoxins, predominantly in the dried fruits and seeds, including pistachios, and the most prevalent group are aflatoxins, followed by ochratoxin A. Above 90% of notifications are from countries outside EU, particularly, Turkey and Argentine [27]. In pistachio nuts between January 2020 and June 2021 RASFF already collected 84 notifications mostly from Turkey, Iran and USA related with aflatoxins and 1 notification concerning ochratoxin A (32.8 µg/kg) in pistachio from USA [28,29].

The goal of this paper was to develop and validate a multi-mycotoxins UHPLC-ToF-MS method to determinate aflatoxins (AFB1, AFB2, AFG1 and AFG2), ochratoxin A (OTA), fumonisins (FB1 and FB2), zearalenone (ZEA), toxin T2 (T2) and toxin HT-2 (HT2) in pistachio nuts. Another purpose of this paper was to compare different sorbents (e.g., EMR-Lipid and Z-Sep) and sorbent mixtures (with different sorbents and ratios) in the d-SPE clean-up step of QuEChERS applied in pistachio, checking matrix effect, recovery, limit of detection (LOD) and limit of quantification (LOQ). Lastly, the optimized method was applied to pistachio market samples.

## 2. Results and Discussion

### 2.1. Extraction and Clean-up Optimization

#### 2.1.1. Optimization of Acidification of Water

**Figure 1:**
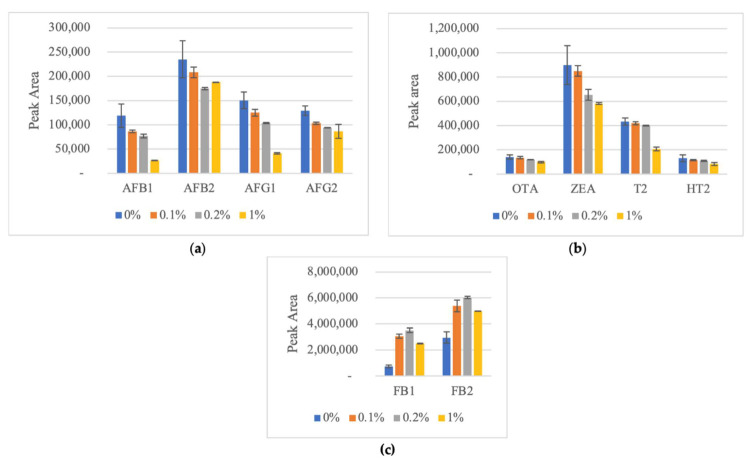
Effect of different levels of acidification of water with formic acid on the extraction of (**a**) AFs (AFB1–Aflatoxin B1; AFB2–Aflatoxin B2; AFG1–Aflatoxin G1; AFG2–Aflatoxin G2) and (**b**) Ochratoxin A (OTA), Zearalenone (ZEA), toxin T2 (T2) and toxin HT-2 (HT2) and (**c**) FBs (Fumonisins, FB1, and FB2) on average peak areas (n = 2).

QuEChERS method was used for the extraction of mycotoxins from pistachio nuts. The procedure involved the extraction of 5 g pistachio with 10 mL acetonitrile after shaking the sample with 10 mL of water acidified with 0.1% of formic acid (FA). In fact, acetonitrile/water extraction (in different percentages) is one of the most common mixtures used for mycotoxin analysis in nuts because solubility of lipids in acetonitrile is limited, thus lipid co-extraction with this solvent is relatively low. In addition, ACN is compatibility with the chromatographic applications [21]. Different amounts of formic acid (0%, 0.1%, 0.2% and 1% *v*/*v*) in water were tested to assure the best results. For these tests, blank samples of pistachio were spiked with 1 ml of calibration work solution, resulting on 2 μg/kg of aflatoxin B1 (AFB1), 4 μg/kg of aflatoxin B2 (AFB2), aflatoxin G1 (AFG1), aflatoxin G2 (AFG2), 3 μg/kg of ochratoxin A (OTA) and 200 μg/kg of fumonisin B1 (FB1), fumonisin B2 (FB2), zearalenone (ZEA), toxin T2 (T2) and toxin HT-2 (HT2). Results show that for AFs, the major peak areas are achieved using pure water (Figure 1). With addition of FA, peak areas reduce, and the lower areas are obtained using 1% FA. The same conclusion was observed for OTA, ZEA, T2 and HT2. Contrary, major peak areas for fumonisins (FB1 and FB2) are achieved with 0.2% of FA, however, using 1% of FA reduced peak area, suggested that fumonisins needs only slight acidification (Figure 1). In conclusion, for optimal results in multi-mycotoxins analysis, addition of 0.1% of FA in water was used in this study, performing the best results, because fumonisins need acidification [30] but other mycotoxins have similar peak areas with pure water or 0.1% FA. In fact, 0.1% of formic acid to extract mycotoxins from pistachio samples has previously been reported in the literature [25,31,32].

#### 2.1.2. Influence of C18, PSA and Z-Sep Sorbents 

**Figure 2:**
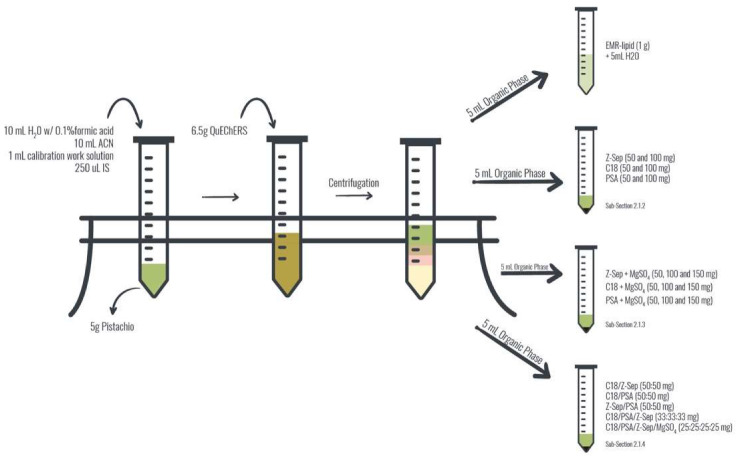
Diagram of different dispersive solid-phase extraction (d-SPE) clean-up procedure experiments.

**Figure 3:**
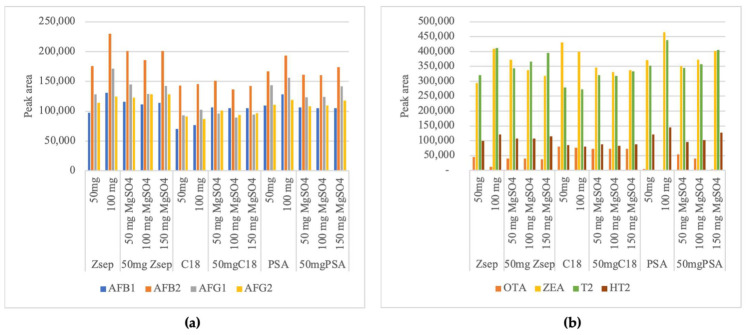
Average peak areas (n = 2) using silica gel with zirconium oxide (Z-Sep), octadecyl modified silica (C18), primary secondary amine (PSA), and magnesium sulfate (MgSO_4_) sorbents in d-SPE clean-up of (**a**) AFs (AFB1–Aflatoxin B1; AFB2–Aflatoxin B2; AFG1–Aflatoxin G1; AFG2–Aflatoxin G2) and (**b**) Ochratoxin A (OTA), Zearalenone (ZEA), toxin T2 (T2) and toxin HT-2 (HT2).

In this study, different clean-up sorbents were evaluated, namely the C18, PSA, Z-Sep and MgSO_4_ in different proportions and mixtures, and EMR-Lipid, using a 5 mL of pistachio extract from QuEChERS spiked with 1 mL of calibration work solution (Figure 2). Conclusions about clean-up efficiency were based on the peak area of each mycotoxin. Results for the single use of sorbents show that, in generally, using 100 mg PSA or Z-Sep result in greater peak areas then 50 mg (Figure 3). Exception is the use of 100 mg C18 that increased peak areas of AFB1, AFB2 and AFG1 when compared with 50 mg, but decrease peak areas for AFG2, FBs, OTA, ZEA, T2 and HT2. This increasing analytical performance using increase amounts of C18 and PSA sorbents was also reported by Zhao et al. [33], and the best results are achieved using 200 mg of C18 for 16 mycotoxins in vegetable oils. Using PSA and C18 caused a significant loss in the analytical signal of OTA, especially using 100 mg of PSA where OTA is not detected. In other way, using 100 mg PSA originated greater signal for ZEA, T2 and HT2 among traditional sorbents.

#### 2.1.3. Influence of Addition of Magnesium Sulfate with C18, PSA and Z-Sep Sorbents 

Magnesium sulfate (MgSO_4_) has been used in clean-up to remove H_2_O [21]. Then, 50, 100 and 150 mg of MgSO_4_ was mixed with 50 mg sorbents. For AFs, addition of MgSO_4_ to C18 results in slight increase of peak area. Regarding PSA, only addition of 150 mg MgSO_4_ give better analytical signals for AFB2, AFG2, OTA, ZEA, HT2 and T2. Addition of MgSO_4_ to Z-Sep give better analytical signals for all mycotoxins comparing to 50 mg Z-Sep, except for OTA. Still, using 100 mg of sorbents is always a better option, despite that for AFB2 the addition of 100 or 150 mg MgSO_4_ and, for T2 addition of any quantity of MgSO_4_ give better analytical signs even better than 100 mg of Z-Sep or 100 mg of C18, respectively. This small increase in peak areas could be less noteworthy because to compare all sorbents, procedure with EMR-Lipid was considering the standard and this method have an additional “polish step” with MgSO_4_ and NaCl (4:1 *w*/*w*) for water removal.

#### 2.1.4. Influence of the Combination of Different Sorbents

**Figure 4:**
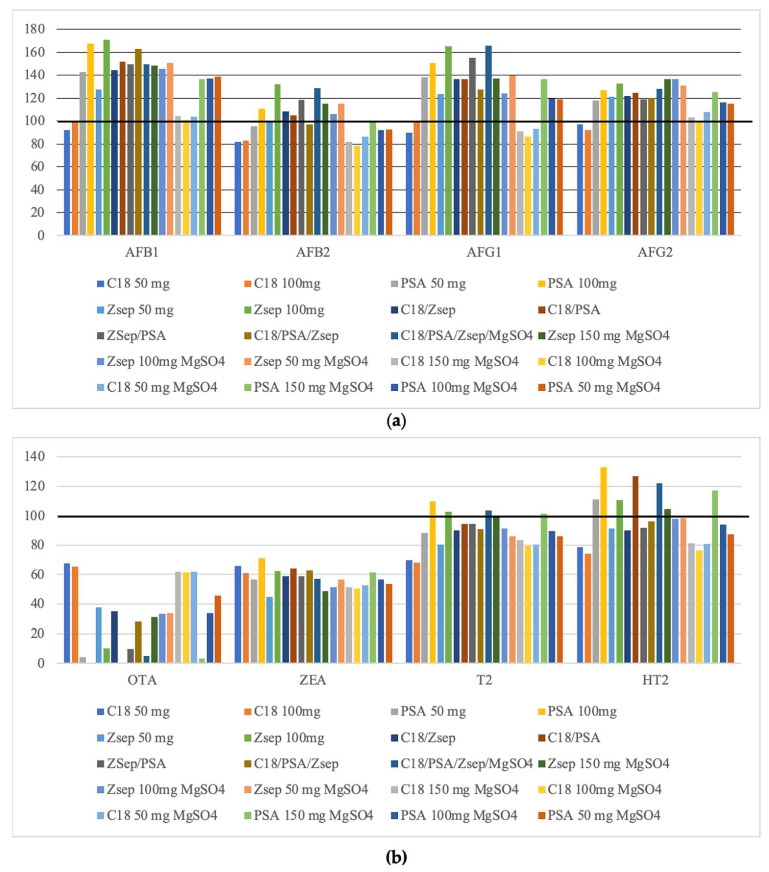
Effect of different sorbents in dispersive solid-phase extraction (d-SPE) clean-up, considering peak areas from enhanced matrix removal-lipid (EMR-Lipid) for comparison (100%) of (**a**) AFs (AFB1–Aflatoxin B1; AFB2–Aflatoxin B2; AFG1–Aflatoxin G1; AFG2–Aflatoxin G2) and (**b**) Ochratoxin A (OTA), Zearalenone (ZEA) and toxin HT-2 (HT2).

Yet, combination of different sorbents in the same proportion was tested (Figure 4). For AFs and T2, mixture of C18, PSA, Z-Sep and MgSO_4_ (25:25:25:25 *w*/*w*) presented the major peak areas. The combination of C18: Z-Sep presents the best analytical signal for OTA, ZEA and HT2.

Among all combinations assayed and considering EMR-lipid peak area as 100% to compare with other sorbents, the results given in Figure 3 showed that better analytical signals were achieved when the 100 mg Z-Sep was used as sorbent for d-SPE. In fact, multi mycotoxins methods are a challenge because mycotoxins have different chemical proprieties, resulting in differences in peak areas and consequent concentration with different sorbents. For mycotoxins, excepting AFs, EMR-Lipid provides the highest analytical signals. Given these results, 1 g of EMR-lipid and 100 mg of Z-Sep were selected to perform clean-up step in mycotoxins analysis.

Solid phase extraction (SPE) on two different C18 cartridges (500 mg and 1 g of C18) was also tested for clean-up. Results show 1 g C18 cartridges increase peak areas from all mycotoxins when compared with 500 mg of C18, however, analytical signal was always lower than EMR-Lipid clean-up. Also, OTA and FBs are present in second elution using 1 g C18, and ZEA and T2 are present in second elution of both columns. It is important to mention that SPE method needs vacuum, use more solvents to condition of column and elution of analytes, and is difficult to apply on large number of samples, so, d-SPE have more advantages because is faster and cheaper [21].

**Figure 5:**
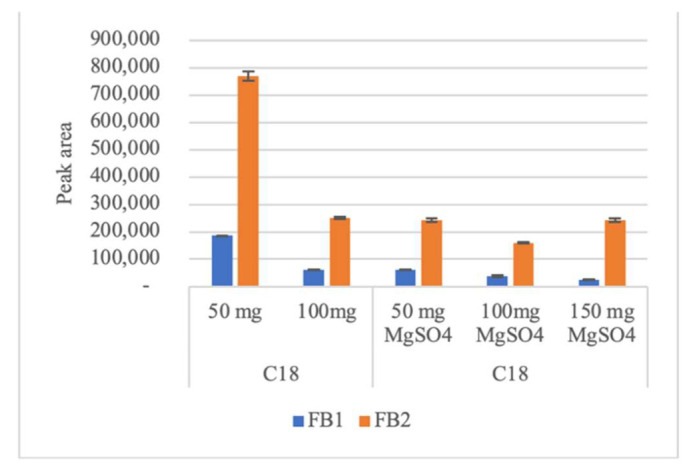
Average peak areas (n = 2) using octadecyl modified silica (C18) and magnesium sulfate (MgSO_4_) in dispersive solid-phase extraction (d-SPE) for Fumonisins (FB1 and FB2).

Concerning fumonisins, using PSA or Z-Sep there is no signal for FB1. Although EMR-Lipid gives better analytical signal for both fumonisins, 50 mg of C18 also give good analytical signal. The use of 100 mg of C18 or the addition of MgSO_4_ decrease peak areas (Figure 5). In the study carried out by Jo et al. [34] in feedstuffs, fumonisins B1 and B2 were also not detected by PSA, while C18 provides analytical signal for all 13 mycotoxins tested.

**Figure 6:**
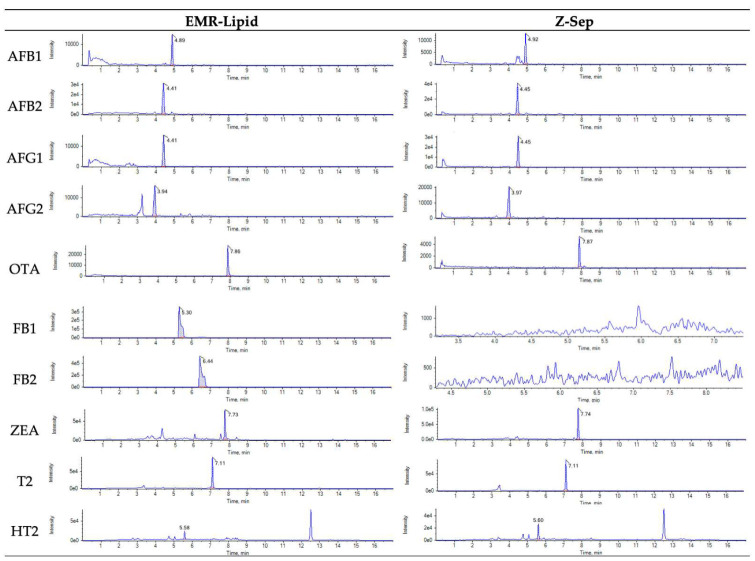
Chromatograms of blank pistachio sample spiked with 2 μg/kg of Aflatoxin B1 (AFB1), 4 μg/kg of Aflatoxin B2 (AFB2), Aflatoxin G1 (AFG1) and Aflatoxin G2 (AFG2), 3 μg/kg of Ochratoxin A (OTA), and 200 μg/kg of Fumonisin B1 (FB1), Fumonisin B2 (FB2), Zearalenone (ZEA), toxin T2 (T2) and toxin HT-2 (HT2), with enhanced matrix removal-lipid (EMR-lipid) and zirconium oxide (Z-Sep) sorbents in dispersive solid-phase extraction (d-SPE) clean-up.

In some studies, for determination of mycotoxins in pistachio nuts, d-SPE clean-up step is not applied because, according to authors, clean-up step reduce the number of mycotoxins analyzed [15,30]. However, there is a decrease in sensitivity, with higher LOQs. Extract clean-up is important to reduce co-extracts which can negatively affects LC-MS/MS equipment and could rapid degradation of the analytical performance of column [19]. D-SPE with EMR-lipid or Z-Sep provides chromatogram with lower background levels as show in Figure 6.

### 2.2. Validation of Analytical Method

Linearity was evaluated by matrix matched calibration curves in different ranges for different mycotoxins (Table 1). Determination coefficients (r^2^) of calibration curves were always higher than 0.99, indicating suitability to quantify mycotoxins in the selected calibration range for both methods. However, determination coefficient was higher for AFB1, AFG1, AFG2, ZEA, T2 and HT2 when Z-Sep is used as sorbent in clean-up step, especially for AFB1 (r^2^ = 0.9993) and ZEA (r^2^ = 0.9994).

The sensitivity of the method was expressed as LOD and LOQ and results are compiled in Table 1. LOD and LOQs are much lower than the requirement imposed by EU regulations for the ML of aflatoxins (AFs and AFB1) in pistachio and sensitive enough to detected other mycotoxins not regulated for nuts. Z-Sep clean-up method provide more sensitivity, LODs and LOQs are lowest, especially for AFs. For ZEA, T2 and HT2, LODs and LOQs are the same for both methods.

LOQs are lower than those reported by Narváez et al. [32] for AFB1, AFB2, AFG2 (0.39 μg/kg) and for AFG1, T2 and HT2 (0.78 μg/kg) using C18 for clean-up followed by UHLPC-Q-Orbitrap MS and lower than those reported by Cunha et al. [19] for AFs (1.25 μg/kg) and for OTA (5 μg/kg) using C18 and Z-Sep^+^ for clean-up followed by HPLC-Quattro Micro triple quadrupole-MS. Our results only indicate higher LOQ for T2 and HT2 than Cunha et al. [19] (1.25 μg/kg). The same EMR-Lipid method with nano flow HPLC-MS allows lowest LOD, for example, 0.05 μg/kg for AFG1, AFG2 and ZEA; 0.5 μg/kg for AFB1, AFB2, FB1 and OTA and 5 μg/kg for FB2, T2 and HT2 [25]. However, regarding OTA, EMR-Lipid method is more sensitivity with LOD of 0.19 μg/kg and LOQ of 0.38 μg/kg.

Table 2 shows the results of recovery, repeatability, and precision inter-day for the different mycotoxins in a blank pistachio sample spiked at 7 different concentration levels. The results regarding the validation of method (Table 2), show that for some mycotoxins there is not linear range when using all the 7 spiking levels, because LODs are higher due to signal-to-noise ratio, or at higher concentration levels there is a loss of linearity. Concerning recovery, Z-Sep provides good recoveries for all mycotoxins within the appropriated range established by the Commission Regulation EC No. 401/2006, ranging between 78 to 119%. These recoveries are comparable to other studies, for example, the recoveries reported by Cunha et al. [19] using C18 and Z-Sep+ in clean-up step (57–102%) and by Alcantara et al. [25] also using EMR-Lipid (70–120%). For EMR-Lipid method, good recoveries are also achieved ranging 79 to 120%.

Repeatability of the method was evaluated by the Relative Standard Deviation (RSD_r_) for all mycotoxins, using the same sample, same operator in a short time and the values are acceptable considering criteria established by Commission Regulation EC No. 401/2006 [35], ranging between 1.30 to 25.25% and 1.21 to 9.92% for EMR-Lipid an Z-Sep method, respectively, considering the eight validated mycotoxins for both methods, excluding FBs. The highest RSD_r_ is for AFG1 at spiked level of 1.0 μg/kg (25.25%) but this value is in accordance with criteria established by Commission Regulation EC No. 401/2006. Regarding each mycotoxin, method using Z-Sep has best repeatability for AFB1, AFG1, AFG2, OTA, ZEA and HT2 and similar repeatability to EMR-lipid for T2. However, for AFB2 the best repeatability was achieved with EMR-Lipid.

Precision inter-day of the method was evaluated by the Relative Standard Deviation (RSD_R_) at 3 different days of analysis, 2 or 3 different concentration levels with different operators and the values are acceptable, ranging between 2.8 to 26.8% and 1.8 to 9.7%, for EMR-Lipid and Z-Sep method, respectively. For precision inter-day, clean-up using Z-Sep presented the best results for all mycotoxins, except for AFB2.

**Figure 7:**
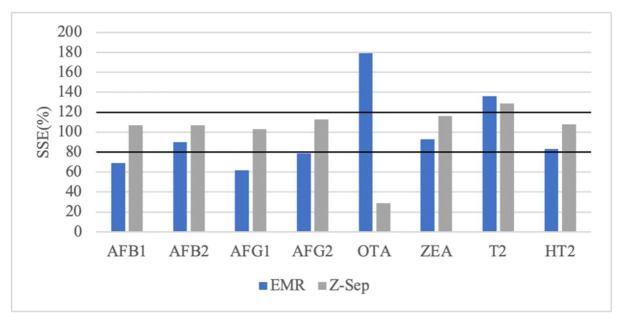
Matrix effect with enhanced matrix removal-lipid (EMR-lipid) and zirconium oxide (Z-Sep) as dispersive solid-phase extraction (d-SPE) sorbents for ten mycotoxins.

Matrix effect (ME) is caused by the alteration of ionization efficiency of target analytes in the presence of co-eluting compounds, affecting negatively analytical performance [36]. Z-Sep cause a signal enhancement for all mycotoxins, excepting OTA (Figure 7). ME was negligible for AFB1(SSE = 107.0%), AFB2 (SSE =107.1%), AFG1 (SSE = 103.3%) and HT2 (SSE = 108.1%) in Z-Sep clean-up, soft to AFG2 (SSE = 112.5%) and ZEA (SSE = 116.1%), and medium to T2 (SSE = 128.7%). For seven mycotoxins, Z-Sep gives the lowest matrix effect varying between negligible to medium (103 to 129%). This signal enhancement in AFs was found by Hidalgo-Ruiz et al. [37] in pistachio using C18 (ME = 42–67%).

However, EMR-Lipid provided signal suppression for AFB1 (SSE = 69.3%), AFB2 (SSE = 90.1%), AFG1 (SSE = 62.4%), AFG2 (SSE = 79.7%), ZEA (SSE = 93.1%) and HT2 (SSE = 83.5%), only for HT2 are a medium signal enhancement (SSE = 136.3%). However, in Alcántara-Durán et al. [25] study, EMR-Lipid sorbent displays negligible matrix effect in all mycotoxins in pistachio samples (between 0 to 6%) It was noticed that, after the d-SPE clean-up step, Z-Sep sorbent gave a greener extract comparing to EMR-Lipid sorbent with yellow tone, so using Z-Sep as sorbent indicate a higher amount of pigment remained in the extract.

In case of OTA, it was a found a strong matrix effect, higher than 50%, using both sorbents, although Z-Sep provides a signal suppression (SSE = 28.8%) and EMR-Lipid a signal enhancement (SSE = 179.3%). The same strong matrix effect for OTA was reported by Cunha et al. [19] using 50 mg C18 and 50 mg Z-Sep^+^ as sorbents in nuts samples, ranging between 174.9 to 231.0%. Similar results are obtained by Arroyo-Manzanares et al. [38] who found ME of −65.6% applying dispersive liquid–liquid microextraction (DLLME) in edible nuts SSE of 194.1% in maize by Sanches Silva et al. [11] and SSE = 180% in vegetable oils using C18 by Zhao et al. [33].

Comparing methods for fumonisins (FB1 and FB2) is not possible because there is only analytical signal for clean-up with EMR-Lipid. The correlation coefficient is good (r^2^ > 0.99) between 25.0 to 200.0 μg/kg and 12.5 to 200.0 μg/kg, for FB1 and FB2, respectively. This method has good recovery (94.4 to 104% for FB1 and 91.1 to 103.5% for FB2), with good values for repeatability (2.2 to 5.7% for FB1 and 1.0 to 8.3% for FB2) and precision inter-day (between 2.6 and 4.56%) (Table 2). EMR-Lipid sorbent causes a strong signal enhancement, this fact as already reported by in maize samples with SSE = 123.6% [39] and SSE = 125.4% [11].

Numerous multi-mycotoxin methods for pistachio, nuts and other foodstuffs based on QuEChERS methodology have been published [25,31,32,34,37,38,40,41,42]. The main difference among those methods is the clean-up step using Immunoaffinity Chromatographic Columns (IACs) or d-SPE with different mixtures of sorbents. IACs is very sensitive and selective technique due to specific of antibodies to mycotoxins, but (1) uses more solvents in washing and elution steps; (2) there is a possibility of cross antibody reaction; and (3) it depends on the availability of columns in the market concerning mycotoxins and matrices. In d-SPE, other authors include a freezing step that increases the time of analysis [41,42] or uses more than one sorbent which has a higher cost [34,40,42]. Recently, some methods are based on “diluted and shoot” approach, a “no clean-up” technique that could affect the performance of the chromatographic equipment [41]. So, QuEChERS with d-SPE using Z-Sep as sorbent, is a simple, rapid and easy technique for application to large number of samples in a short time, with less use of reagents, solvents and materials, allowing effective extraction of mycotoxins and removing lipids and other compounds present in pistachios that can interfere with the HPLC system.

Also, this validated UHPLC-ToF-MS method provides high sensitivity and specificity for identification, quantification and confirmation of multi-class mycotoxins, where identification of molecules is based on molecular weight. This MS detector has advantages when compared, for example, with previously used fluorescence detection (FLD) [14,43,44,45,46,47] which is only applicable for AFs due to their fluorescent properties (AFB1 and AFB2 exhibit fluorescence at 425 nm, AFG1 and AFG2 exhibit fluorescence at 450 nm) [48] and required a derivatization step to increase resolution and sensitivity [49,50].

It is also important to refer that QuEChERS protocol involving EMR-Lipid sorbent was more time-consuming due to the two extra steps to active sorbent with water to achieve better efficiency and then step with MgSO_4_ and NaCl to obtain a phase separation between H_2_O and ACN [24]. In this study, to better compare between EMR-Lipid and Z-Sep sorbents, this second extra step was also applied, but in literature using Z-Sep there is no need [24].

So, it could be concluded that Z-Sep sorbent is the most efficient way to remove matrix interferents matrix, easier and faster, providing best analytical performance for multi-mycotoxins method ranging AFs, ZEA, T2 and HT2. However, for OTA, EMR-Lipid is the best option for clean-up due to the lowest LOD and LOQ and since mycotoxins are present at low concentration in pistachio, Z-Sep provides better precision (repeatability and precision inter-day). For fumonisins, Z-Sep is not a good sorbent. In this case, EMR-Lipid provide good analytical performance.

It should be highlighted that although no legal limits have been defined for mycotoxins other than AFs in pistachio, climate changes have an impact on abiotic factors as temperature, water activity (a_w_), relative humidity, and CO_2_, known as critical factors to fungal growth and mycotoxins’ production in field and/or during storage [51,52,53,54]. Due to this fact, new multi-mycotoxin methods for determination of mycotoxins in pistachio nuts should be validated, in order to detect simultaneously more mycotoxins to ensure food safety.

### 2.3. Occurrence of Mycotoxins in Pistachio

In order to show the applicability of the method, sixteen samples of pistachio were analyzed using Z-Sep as sorbent for d-SPE clean-up and also using EMR-Lipid to determine fumonisins. Each sample was extracted in duplicate.

**Figure 8:**
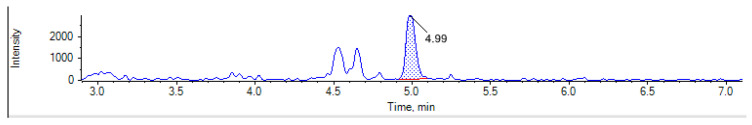
Chromatogram of pistachio kernel sample contaminated with Aflatoxin B1 (AFB1).

One sample, corresponding to a raw pistachio from Iran, was detected with 0.20 μg/kg of AFB1 (Figure 8). It should be noted that this concentration is following the current ML established by the EU for aflatoxins in nuts. Alcántara-Duran et al. [25] also detected one sample with AFB1, but above LOQ and Liao et al. [55] report two samples with 0.5 μg/kg and 1.2 μg/kg of AFB1.

AFB2 was detect in another sample at 0.73 μg/kg, lower than ML established for the sum of AFB1, AFB2, AFG1 and AFG2 (4 μg/kg). Similar results are obtained by Liao et al. [55]. which detected in one out of ten pistachio samples at 0.9 μg/Kg of AFB2 In this study, pistachio shells were analyzed and in two samples AFB2 was quantified (0.53 and 0.56 μg/kg).

**Figure 9:**
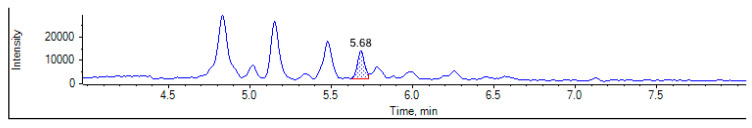
Chromatogram of pistachio kernel sample contaminated with HT-2 toxin (HT2).

HT2 was found in three samples at 50.63 μg/kg, 67.37 μg/kg and 71.56 μg/kg (Figure 9). These pistachios samples are in bulk sale, indicating that temperature and/or relative humidity conditions are not optimum to storage. HT-2 toxin is produced by species *Fusarium sporotrichioides* and *F. poae*, and mostly found in oats, corn and wheat [56], so no limits are established at EU for this mycotoxin in nuts.

Fumonisin B1 was detected in one pistachio kernel and shell from USA, but at a concentration lower than the LOQ (25 μg/kg). However, no limits are established for fumonisins in nuts. Fumonisins are produced by *Fusarium proliferatum* and *F. verticillioides*, and predominantly found in corn and derived products [56].

Various studies reported occurrence of mycotoxins in pistachio nuts. Aflatoxins, especially AFB1, and OTA are the most frequently detected, and none of the studies from the last 2 decades show contamination with FBs, ZEA, T2 or HT2.

In the EU, pistachio samples collected from Italy markets by Diella et al. [43] showed median level of 31.9 μg/kg for AFB1 and from Catalonia (Spain) by Coronel et al. [57] results showed a mean concentration of 0.228 μg/kg for OTA. Cheraghali et al. [20] found 37% samples from Iran were contaminated with AFB1 and 11.8% were above maximum permitted levels in Iran (5 μg/kg) [14], which is higher than that established permitted levels at EU.

## 3. Materials and Methods

### 3.1. Chemicals and Reagents

Methanol, acetonitrile (ACN), both HPLC gradient grade, and formic acid were purchased from Merck (Darmstadt, Germany). Water was purified by Milli-Q plus system from Millipore (Molsheim, France) with resistivity of 18.2 MΩ × cm. Mycotoxins standards and internal standard (zearalenone, ZAN) were purchased from Sigma–Aldrich (Madrid, Spain) and were dissolved in acetonitrile (AFB2, AFG1, ZEA, T2 and ZAN), methanol (AFB1, AFG2 and OTA) or acetonitrile:water (50:50, *v*/*v*) (FB1 and FB2). Stock solutions were prepared with a concentration of 1 mg/mL, except T2, which presented a concentration of 25 mg/mL. These stock solutions were subsequently used to prepare working solution for calibration. Calibration work solution were prepared in acetonitrile with concentration of 10 ng/mL of AFB1; 20 ng/mL of AFB2, AFG1 and AFG2; 15 ng/mL OTA and 1 μg/mL of FB1, FB2, T2, HT2 and ZEA. All standard solutions were stored in amber vials in the dark at –20 °C, for at least 2 years [11], and before use, they were kept at room temperature for 15 min.

For QuEChERS, trisodium citrate dihydrate and anhydrous magnesium sulfate were purchased from PanReac (Barcelona, Spain). Sodium chloride was purchased from Fluka (Seelze, Germany). Sodium citrate dibasic sesquihydrate was purchased from Sigma-Aldrich (Madrid, Spain). For clean-up procedures, EMR-Lipid d-SPE tubes were purchased from Agilent Technologies (Santa Clara, CA, USA) and Z-Sep from Supelco-Merck (Darmstadt, Germany). For clean-up tests, primary secondary amine-bonded silica (PSA) and C18 were acquired from Agilent Technologies (Santa Clara, CA, USA). Sep-Pak columns of C18 (1 g and 500 mg) were purchased from Waters (Woods Hole, MA, USA).

### 3.2. Samples and Sampling Procedure

Sixteen samples of pistachio nuts (raw or roasted, salted or natural, conventional and biological products, packaged and bulked) were randomly purchased in different supermarkets in Portugal between February and April of 2021 for determination of mycotoxins. Samples are from Iran, United States of America, and Spain. In-shell pistachios were pealed. Pistachio kernels and pistachio shells samples (500–1000 g) were ground (Retsch rotor mill SK 300 with a sieve of trapezoid holes of 1.00 mm), mixed thoroughly to assure complete homogenization and preserved at −20 °C until analysis.

### 3.3. Extraction Procedure

Mycotoxin extraction was performed according to a QuEChERS procedure: about 5 g of pistachio (5.0 ± 0.1 g) was weighted in 50 mL polypropylene tubes. First, 250 μL at 10 μg/mL of zearalanone (ZAN) was added. Afterward, samples are hydrated with 10 mL of ultrapure water with 0.1% of formic acid and 10 mL of acetonitrile is added. Then, the sample and the extractant was mixed for 1 min in vortex. Next, mixture of extraction salts for liquid–liquid partitioning step (4 g of anhydrous magnesium sulfate, 1 g of sodium chloride, 1 g of sodium citrate and 0.5 g of disodium hydrogen citrate sesquihydrate) were added and mixed for 1 min in vortex, following by centrifugation at 12,669× *g* for 5 min at 5 °C. Finally, organic phase was used to carry out the d-SPE procedure, testing different sorbents:

**Experiment 1:** EMR sorbent in 15 mL falcon tube was first activated with 5 mL of ultrapure H_2_O and vortexed for 30 s. After, 5 mL of organic extract were added, vortexed for 1 min and then centrifuged at 12,669× *g* for 5 min at 5 °C. Then, supernatant was decanted for 15 mL falcon tube with 1.6 g of anhydrous magnesium sulfate and 0.4 g of sodium chloride to obtain a phase separation between H_2_O and ACN, followed by vortex for 1 min and centrifugation at 12,669× *g* for 5 min at 5 °C. Afterwards, 4 mL of the extract was transferred to a 15 mL falcon tube and evaporated to dryness under a gentle stream of nitrogen at 40 °C.

**Experiment 2:** 5 mL of organic phase were transferred into a 15 mL falcon tube with 100 mg of Z-Sep. The mixture was shaken for 1 min in vortex and then was centrifuged at 12,669× *g* for 5 min at 5 °C. To compare with EMR-Lipid procedure, supernatant was decanted for 15 mL falcon tube with 1.6 g of anhydrous magnesium sulfate and 0.4 g of sodium chloride, followed by vortex for 1 min and centrifugation at 12,669× *g* for 5 min at 5 °C. After, 2 mL of the extract was transferred to a 15 mL Falcon tube and evaporated to dryness under a gentle stream of nitrogen at 40 °C.

Finally, residues from EMR-lipid and Z-Sep d-SPE procedure were redissolved with 500 μL of acetonitrile 40% (*v*/*v*), vortexed for 30 s follow by 15 min in an ultrasonics bath and filtered through a PVDF mini-uniprep™ for injection into the UHPLC-ToF-MS system.

#### 3.3.1. Clean-up Experiments

Different clean-up sorbents were evaluated, namely the C18, PSA, Z-Sep and MgSO_4_ in different proportions and mixtures, and EMR-Lipid, using a 5 mL of pistachio extract from QuEChERS spiked with 1 mL of calibration work solution (Figure 2).

#### 3.3.2. Spiking Experiments

To determine the recovery of the target analytes, spiking experiments were performed. The matrix-matched calibration was prepared by spiking blank sample of pistachio (5 g) with 7 different levels, using 0.0625 mL to 2 mL of calibration of the work solution (sub-Section 3.1) to obtain a concentration range between 0.125 to 4.0 μg/mL of AFB1; 0.250 to 8.0 μg/mL of AFB2, AFG1 and AFG2; 0.19 to 6.0 μg/mL of OTA; 12.5 to 400.0 μg/mL of FB1, FB2, ZEA, T2 and HT2. Subsequently, extraction was performed as described in sub-Section 3.3. This concentration levels include the maximum levels imposed for mycotoxins in EC Regulation No. 1881/2006 for nuts [16]. Even though there is no legislation for *Fusarium* mycotoxins (FBs, ZEA, T2 and HT2) in nuts, there is an EC Recommendation of 27 march of 2013 for the presence of T-2 and HT-2 toxins in cereals and cereal products [58] and EC Regulation No. 1881/2006 establishes maximum levels for ZEA and FBs for cereals for direct human consumption [16]. For validation purposes, the concentration range considered was 12.5 to 400 μg/kg to include all the levels found EC Regulations and Recommendations for cereals, because we considered cereals as a possible reference to pistachio nuts due to the fact that both matrices are solid with some similarity in water composition (raw pistachios: < 5% water [59] and cereals: mean of 12% [60,61,62,63,64]), although lipid content is higher for pistachio.

Before method development, 3 different pistachio samples were analysed to ensure that any mycotoxin would not be present, using modify method based on Sanches Silva et al. [11] method with two-step extraction with acetonitrile 80% (*v*/*v*). Thus, the selected blank samples were analysed by this new method, and none of the studied mycotoxins were detected.

#### 3.3.3. Matrix Effect

To evaluate the influence of co-extracted compounds on analytical signals, the matrix effect (ME) was determined by the signal suppression-enhancement (SSE), comparing the slope of calibration standard solution and slope of matrix-matched calibration curve with fortified pistachio samples. Signal enhancement was considered when SSE > 100%, inexistence of the matrix effect when SSE = 100% and signal suppression when SSE < 100%. According to several authors [25,65], matrix effect could be classified as negligible ([0%]–[±10%]), soft ([±10%]–[±20%]), medium ([±20%]–[±50%]) and strong ([±50%]).

### 3.4. UHPLC–ToF-MS Parameters

Detection and quantification were performed with a Nexera X2 Shimadzu UHPLC coupled with a 5600+ ToF-MS detector (SCIEX, Foster City, CA, USA) equipped with a Turbo Ion Spray electrospray ionization source working in positive mode (ESI+). In terms of chromatographic conditions, a column Zorbax Eclipse Plus C18 (2.1 mm × 50 mm, 1.8 μm) was used and kept at 30 °C, the autosampler was maintained at 10 °C to refrigerate the samples and a volume of 20 μL of sample extract was injected in the column. The mobile phase consisted of 0.1% formic acid [a] and acetonitrile [B] with a flow rate of 0.5 mL/min and with the following gradient program: 0–12 min from 90% to 30% [A]; 12–13 min from 30% to 10% [A] and kept until 14 min; back to 90% [A] from 14 to 15 min until the end of the run. The total run time was 17 min. In terms of mass spectrometry, the acquisition was performed in full-scan from 100 to 750 Da using the Analyst^®^ TF software (SCIEX, Foster City, CA, USA) and with the following settings: ion source voltage of 5500 V; source temperature 575 °C; curtain gas (CUR) 30 psi; Gas 1 and Gas 2 of 55 psi; declustering potential (DP) of 100 V. Every 7 injections the ToF-MS detector was calibrated in the mass range of the method, to guarantee the accurate mass resolution.

### 3.5. Identification of Mycotoxins

The identification and data processing of mycotoxins were made through the PeakView™ and MultiQuant™ software (SCIEX, Foster City, CA, USA).

The isotope match is presented automatically by the PeakView™ software, and regarding identification criteria of mycotoxins, three parameters and their corresponding equations (Equations (1)–(3)) were used: (1) maximum relative retention time deviation (ΔRRT) of 2.5% (Equation (1)); (2) difference in the isotope pattern with a tolerance of 10% (Equation (2)); and (3) exact mass deviation (Δm) with a tolerance of 5 ppm (Equation (3)).

(1) $$RRT=\frac{RT_{analite}}{RT_{internal\;standard}}$$

where *RT_analite_* is the retention time of *analite*, and the *RT_internal standard_* is the retention time of internal standard (zearalanone).

(2) $$RRT=\;\left(\frac{RRT_{spiked\;samples}-RTT_{standard}}{RRT_{standard}}\right)\;\times \;100$$

(3) $$\Delta m\;\left(ppm\right)=\;\left(\frac{Exact\;mass-Detected\;mass}{Exact\;mass}\right)\times 10^{6}.$$

### 3.6. Validation of the UHPLC-ToF-MS Method

The method was validated by the determination of concentration range, linearity, limit of detection (LOD), limit of quantification (LOQ) and accuracy by determining precision (repeatability and precision inter-day) and trueness by recovery assays at different levels). According to Decision of 12 August 2002 implementing Council Directive 96/23/EC concerning the performance of analytical methods and the interpretation of result, when certified reference materials are not available, trueness of measurements can be assessed through recovery of additions of known amounts of the analytes to a blank matrix [66].

LOD and LOQ were determined as the concentration that originates a signal-to-noise ratio (S/N) ≥3 and ≥10, respectively. For the determination of repeatability (RSD_r_) and precision inter-day (RSD_R_), blank samples of pistachio were spiked at different levels (n = 6) take in account the ML of each mycotoxin. In the case of RSD_R_ extraction was carried out in three different days by two different operators.

## 4. Conclusions

An analytical method based on QuEChERS followed by ultra-high-performance liquid chromatography coupled with high-resolution mass spectrometry was validated for the simultaneous detection of eight mycotoxins in pistachios.

For matrices with high lipid content, like pistachio nuts, it becomes evident that the clean-up step is fundamental for reducing interferences in the analysis and allowing a smaller number of maintenances in analytical equipment. The optimized procedure includes evaluation of different sorbents, and lastly EMR-Lipid and Z-Sep are compared. It was concluded that the use of 100 mg of Z-Sep provided best analytical performance, with good recovery (79 to 120%), repeatability (RSD_r_ < 10%) and precision inter-day (RSD_R_ < 10%) in agreement with criteria established by Commission Regulation EC No.401/2006 for mycotoxins analysis [35]. The LODs for AFs ranged from 0.125 to 0.25 ug/kg, which are lower than the maximum levels in nuts regulated by the EU. Although for OTA, LOD and LOQ are lower using EMR-lipid, precision (RSD_r_ and RSD_R_) is better using Z-Sep. Z-Sep procedure is easier and faster, comparing to EMR-Lipid sorbent which had to be active with water before clean-up. Method with EMR-Lipid sorbent also gives good performance for determination of mycotoxins, including fumonisins, according to criteria in Commission Regulation EC No. 401/2006 [35]. But, considering AFs as the mycotoxins of greatest interest in pistachios, contrary to FBs, Z-Sep sorbent provides more advantages.

In addition, 6 of 16 real samples of pistachios were found to be contaminated with one mycotoxin (AFB1, HT2 or FB1) but at low concentrations. The concentration of AFB1 was lower than legislated. Also, AFB2 and FB1 are detected in pistachio shells. Further studies in pistachio samples from different origin countries should be carried out. Also, this method could be applied in other tree nut fruits and peanuts, which have a higher consumption and play an important role on mycotoxins human exposure.

## Figures and Tables

**Table 1:** Linearity and sensitivity of UHPLC-ToF-MS method for the simultaneous determination of mycotoxins in pistachio.

Mycotoxin	LOD (μg/kg)		LOQ (μg/kg)		Linear Range (μg/kg)		Calibration Curve Parameters					
							r^2^		Slope		Interception	
	EMR	Z-Sep	EMR	Z-Sep	EMR	Z-Sep	EMR	Z-Sep	EMR	Z-Sep	EMR	Z-Sep
AFB1	0.125	0.125	0.5	0.125	0.5−4.0	0.125−2.0	0.9901	0.9993	32491.9	60259.9	4765.9	571.7
AFB2	0.25	0.25	0.5	0.25	0.50−8.0	0.25−4.0	0.9989	0.9973	42436.2	49476.5	−2191.6	1238.5
AFG1	0.50	0.25	1.0	0.25	1.0−8.0	0.25−4.0	0.9929	0.9974	17067.9	38603.0	11613.0	−967.9
AFG2	0.50	0.25	1.0	0.25	1.0−4.0	0.5−8.0	0.9931	0.9976	22299.4	30307.8	6944.3	2158.1
OTA	0.19	0.75	0.38	1.50	0.38−3.0	1.5−6.0	0.9934	0.9914	32398.2	6792.0	1900.9	−2529.1
ZEA	12.5	12.5	25	12.5	25−200	12.5−400	0.9958	0.9994	1504.5	1910.7	−3896.5	−1000.2
T2	12.5	12.5	25	25	25−200	25−400	0.9938	0.9979	1644.7	1570.9	12400.9	3811.1
HT2	25	25	25	25	25−400	25−400	0.9976	0.9979	287.6	403.1	7879.4	7252.4
FB1	12.5	*	25	*	25−200	*	0.9961	*	16960.4	*	−33602.9	*
FB2	12.5	*	12.5	*	12.5−200	*	0.9983	*	28671.6	*	−1339.8	*

**LOD**—limit of detection; **LOQ**—limit of quantification; **EMR**—enhanced matrix removal-lipid; **AFB1**—aflatoxin B1; **AFB2**—aflatoxin B2; **AFG1**—aflatoxin G1; **AFG2**—aflatoxin G2; **FB1/FB2**—fumonisins B1 and B2; **OTA**—ochratoxin A; **T2/HT2**—trichothecenes; **ZEA**—zearalenone. * FB1 and FB2 are not detected when using zirconium oxide (Z-Sep) as sorbent.

**Table 2:** Results of the validation for different mycotoxins, including recovery (Rec), relative standard deviation repeatability (RSDr) and relative standard deviation of precision inter-day (RSDR) at different spiking levels with enhanced matrix removal-lipid (EMR-Lipid) and zirconium oxide (Z-Sep) sorbents in dispersive solid-phase extraction (d-SPE) clean-up.

			EMR-Lipid				Z-Sep			
Mycotoxin	Ion	Retention Time (min)	Spiked Level (μg/kg)	Rec (%) (n = 6)	RSD_r_ (%)	RSD_R_ (%)	Spiked Level (μg/kg)	Rec (%) (n = 6)	RSD_r_ (%)	RSD_R_ (%)
AFB1	313.07066 [M + H]^+^	5.00	0.50 1.0 1.5 2.0 4.0	119.1 89.1 101.9 101.9 100.2	10.68 8.27 10.73 10.35 11.90	3.70 8.80	0.125 0.250 0.50 1.0 1.5 2.0	93.3 98.1 98.9 100.7 97.3 101.4	3.59 6.99 4.97 5.29 5.06 3.59	3.77 2.56
AFB2	315.08631 [M + H]^+^	4.52	0.50 1.0 2.0 3.0 4.0 8.0	111.4 102.6 100.5 100.4 97.9 97.5	4.85 6.16 6.90 2.97 4.83 2.43	3.54 3.56 2.79	0.25 0.50 1.0 2.0 3.0 4.0	102.8 98.0 95.3 99.9 100.3 96.5	8.69 6.09 6.65 5.56 6.68 5.64	5.67 4.12
AFG1	329.06558 [M + H]^+^	4.53	1.0 2.0 3.0 4.0 8.0	77.3 76.9 97.9 104.6 101.7	25.25 24.74 7.70 6.68 12.98	6.76 3.25	0.25 0.50 1.0 2.0 3.0 4.0	105.7 112.0 92.1 101.8 99.3 97.6	5.71 6.83 9.62 6.72 5.04 6.01	8.17 5.62
AFG2	331.08123 [M + H]^+^	4.04	1.0 2.0 3.0 4.0	86.0 95.4 100.1 104.3	20.66 11.78 12.04 7.02	26.75 9.37	0.50 1.0 2.0 3.0 4.0 8.0	119.7 100.4 103.6 96.3 98.0 99.4	5.37 4.82 5.83 9.92 4.10 9.44	9.69 2.96 2.21
OTA	404.08954 [M + H]^+^	7.97	0.38 0.75 1.50 2.25 3.0	95.0 97.5 100.4 108.5 102.3	10.30 11.95 14.74 4.81 10.04	9.85 9.01	1.50 2.25 3.0 6.0	105.9 88.7 109.4 82.6	9.26 6.97 7.31 3.99	7.85 6.35
ZEA	319.154 [M + H]^+^	7.83	25 50.0 100 150 200	93.9 96.3 112.9 92.1 99.6	1.61 10.21 16.38 14.25 5.43	10.05 7.41	12.5 25 50.0 100 150 200 400	106.0 102.4 97.1 94.7 97.1 98.8 98.8	2.56 7.18 4.77 8.54 4.13 5.48 2.74	4.47 2.49 3.97
T2	489.2095 [M + Na]^+^	7.21	25 50.0 100 150 200	80.8 99.1 100.6 106.9 96.7	2.40 1.30 3.41 6.98 3.33	9.42 6.33	25 50.0 100 150 200 400	100.2 97.3 105.6 98.3 98.86 97.9	5.89 3.75 3.38 1.85 5.29 7.24	7.82 7.92 1.77
HT2	425.217 [M + H]^+^	5.69	50.0 100 150 200 400	118.9 90.7 108.3 109.8 102.2	15.23 9.12 11.35 10.79 2.69	10.81 7.42	25 50.0 100 150 200 400	75.2 91.2 105.0 108.2 103.8 99.8	3.89 7.42 6.88 1.21 2.12 3.70	8.89 6.82 7.75
FB1	722.396 [M + H]^+^	5.32	25 50.0 100 150 200	104.0 94.4 99.3 101.9 99.2	4.01 5.36 2.18 5.66 4.65	2.58 4.56	na	na	na	na
FB2	706.401 [M + H]^+^	6.48	12.5 25 50.0 100 150 200	101.3 100.1 91.1 103.5 100.0 99.4	4.67 3.17 8.28 1.02 3.56 2.98	3.66 3.43	na	na	na	na

na—not applicable.

## Data Availability

Data sharing is not applicable to this article.

## Sample Availability

Samples of the compounds are not available from authors.
